# Update in juvenile myositis

**DOI:** 10.1097/01.bor.0000434674.62644.02

**Published:** 2013-09-25

**Authors:** Kiran Nistala, Lucy R. Wedderburn

**Affiliations:** a Centre for Rheumatology, University College London; b Rheumatology Unit, UCL Institute for Child Health, London; c Arthritis Research UK Centre for Adolescent Rheumatology at UCL, UCLH and GOSH, UK

**Keywords:** autoantibodies, B-cell depletion, juvenile dermatomyositis

## Abstract

**Purpose of review:**

This update on childhood idiopathic inflammatory myopathies (IIMs) reviews recent progress in the field of translational science and clinical research over the past 12–18 months.

**Recent findings:**

Several new studies, including results from the international genome-wide association study, point to abnormalities of the adaptive immune system in childhood IIMs. Circulating T-follicular helper cells promote plasma cell differentiation and have been found in high levels in juvenile dermatomyositis (JDM), which may account the frequency of autoantibodies seen in this disease. One of the latest to be identified in JDM targets the protein NXP-2 and is associated with an increased risk of calcinosis in young patients. The first randomized controlled clinical trial in refractory adult and childhood IIMs was reported this year. B-cell depletion with the anti-CD20 antibody, rituximab, failed to achieve its primary end point, but patients with JDM did show good improvement in disease activity. A new international definition of disease remission in JDM has been agreed, which will aid disease assessment in future therapeutic trials.

**Summary:**

The challenges of studying a rare disease such as JDM have been overcome by several collaborative studies and have led to significant progress in understanding the cause, treatment and prognosis of childhood IIMs.

## INTRODUCTION

The childhood idiopathic inflammatory myopathies (IIMs) are a group of rare but serious multisystem diseases of which the most common is juvenile dermatomyositis (JDM), whereas juvenile polymyositis is seen in less than 5% of cases in most cohorts. It is increasingly clear that the childhood IIMs are in fact relatively heterogeneous: recent advances have shed new light on biomarkers and predictors of this heterogeneity. In this review, we will consider novel developments in our understanding of cause as well as new studies of the clinical assessment, treatment and outcomes of the juvenile IIM.

### Cause

Over the past year, there has been significant progress in understanding the cause of childhood IIMs, including completion of an international genome-wide association study of IIM, novel studies linking JDM with environmental factors, and developments in the field of immunology.

#### Genetics

Investigators from MYOGEN, an international myositis genetics consortium, have carried out the first genome-wide association study of adult and paediatric cases of dermatomyositis from across the US and Europe. As well as confirming the known association with human leukocyte antigen (HLA) region, the results have identified several genes not previously linked with IIM [1]. These results offer exciting new avenues for future research, to explore the functional consequences of these genetic polymorphisms and how these may alter the risk of muscle inflammation. In a separate genetics study, Chinoy *et al.*[2] studied the genetic association of the nuclear factor kappa B (NF-KB), a known signalling intermediary in inflammation, with IIMs of both adults and children. An allele of the *inhibitor of kappa B-like* (*IKBL)* gene, which is part of the NF-KB family, was associated with myositis cases when compared to controls, but after controlling for linkage disequilibrium with the HLA 8.1 haplotype, this effect was lost.

**Box 1:**
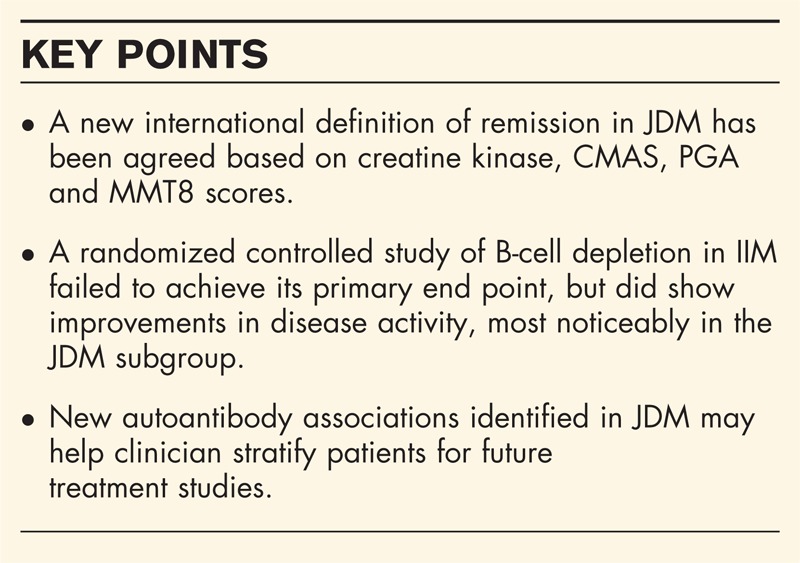
no caption available

The above data reinforce the hypothesis that JDM is a complex genetic disorder with each genetic association having a modest effect on the risk of acquiring JDM. However, the study of monogenic human disorders also offers insights into the cause of JDM; patients with the rare inflammatory disorder, CANDLE syndrome – chronic atypical neutrophilicdermatosis with lipodystrophy and elevated temperature – share clinical and immunological features with JDM, including myositis, lipodystrophy and a prominent interferon (IFN)-α signature in whole blood samples. Mutations in the immunoproteasome, a family of proteins that process degraded intracellular proteins, have been identified as the cause of CANDLE syndrome [3^▪^]. This is of particular interest to IIMs, as several related pathways involved in the trafficking and folding of proteins within the endoplasmic reticulum have already been implicated in pathogenesis of IIMs [4].

#### Environment

It has been a longstanding clinical observation that sunlight can precipitate disease flares in JDM and juvenile systemic lupus erythematous (SLE). A recent study [5] suggests that exposure to ultraviolet (UV) light could also trigger disease onset; the authors found that the intensity of UV light in the month prior to disease presentation increased the risk of developing JDM relative to juvenile polymyositis. This risk was only detectable in patients positive for the p155/140 myositis-associated autoantibodies who represented 30% of this cohort, which is in line with previous studies [6]. This raises the interesting question of how sunlight might promote the breakdown of B-cell tolerance. The target for the p155/140 antibody has been identified as the transcription factor, transcriptional intermediary factor (TIF)-1, a member of the tripartite motif (TRIM) family of proteins [7]. The authors speculate that sunlight may up-regulate interferon type 1 cytokines which secondarily up-regulate the TRIM proteins leading to an autoantibody response.

Although JDM patients are advised to use sun block and avoid sun exposure, this could lead to reduced levels of vitamin D. Vitamin D metabolites are important in inducing tolerogenic dendritic cells [8], so one inadvertent consequence of sun avoidance could be a failure of immunoregulation which will result in increased disease activity. To test this hypothesis, Robinson *et al.*[9] measured 25-hydroxylated vitamin D in 21 JDM patients and found that serum levels inversely correlated with physician's global disease activity scores, after controlling for vitamin D supplementation and race, which are two known influences on vitamin D status. Sadly, vitamin D as a therapy has yet to show significant benefits as a treatment for autoimmune disease [10].

#### Immunity

Researchers have investigated the immunonopathology of childhood IIMs by detecting abnormalities in the peripheral blood compartment and muscle tissue taken during diagnostic procedures.

*Peripheral blood compartment*: It is increasingly clear that the autoantibody status of patients with JDM is strongly associated with distinct clinical phenotypes [6]. However, thus far, it is unclear if autoantibodies represent an epiphenomenon of failed tolerance or themselves contribute to the pathogenic process. To investigate this, Balboni *et al.*[11] screened sera from 36 JDM patients to detect autoantibodies and correlated results with serum interferon alpha activity detected by a functional reporter cell assay. They found that serum with antibodies against Ro, La, Smith and ribonuclearprotein (RNP) had an increased capacity to induce (IFN-α), although they did not test if this was a direct or indirect effect of the circulating autoantibodies.

In a seminal paper, the paediatric rheumatology team at Baylor (Texas, USA) explored the immunological mechanisms that may account for high frequency of autoantibodies in the juvenile IIMs. They identified a subset of circulating T cells, expressing the chemokine receptor CXCR5, which are likely the human equivalent of murine T-follicular helper cells (Tfh) [12]. These human CXCR5+ Tfh support the differentiation of naïve B cells into antibody-producing plasmablasts in an IL-10, inducible T cell co-stimulator (ICOS) and IL-21-dependent manner, and were present at a higher frequency in the blood of patients with JDM when compared with age-matched controls. Moreover, circulating CXCR5+ Tfh correlated with the frequency of plasmablasts in JDM, but only in patients with active disease. The authors did not assess autoantibody status, but it would be interesting to test if Tfh/plasmablasts were particularly enriched in patients with particular myositis-associated autoantibodies.

Other groups have tested the importance of the JDM peripheral blood mononuclear cell compartment in disease pathology, by examining changes in cell frequencies over time and correlating this with alterations in disease activity. Ernste *et al.*[13] found a positive correlation between changes in myeloid dendritic cell compartment and extramuscular disease activity and a negative correlation between the plasmacytoid dendritic cell frequency and muscle disease activity. However, in both cases, correlations were relatively weak, which may reflect the heterogeneity of the patient population or the limitations of using the peripheral blood compartment as a surrogate for inflammatory reactions of the skin or muscle.

*Muscle*: Two new studies examine the mechanisms that control gene expression in myositic tissue. In the first, the authors investigate the regulation of vascular cell adhesion molecule (VCAM)-1, an inflammatory mediator that is enriched in JDM muscle tissue. Previous studies have identified a role for micro-RNA (miR)-126, one of a family of small non-coding RNAs that bind and degrade messenger RNA, in regulating VCAM-1 expression [14]. In muscle biopsies from untreated JDM patients, miR-126 was significantly decreased compared with controls, and these patients had higher muscle VCAM-1 expression [15]. In their second study [16^▪^], the group investigated the epigenetic regulation of muscle gene expression by carrying out whole-genome DNA methylation profiling on muscle tissue from JDM patients prior to treatment. The authors identified 27 genes that were differentially methylated between JDM and control muscle. None of these was linked with the endoplasmic reticulum (ER) stress or type I interferon, pathways known to be involved in JDM. However, six genes within the homeobox family were differentially methylated, including the gene WT1 (whose protein product was first described in association with Wilms tumour), which was significantly hypomethylated in JDM. Consistently with this, WT1 protein levels were significantly increased in JDM muscle, which may play a role in maintaining the pool of pluripotent muscle stem cells in regenerating muscle.

### Diagnosis and clinical assessment

One of the ongoing challenges in the management of JDM has been the difficulty in identifying residual disease activity after treatment. Lazarevic *et al.*[17^▪^] used a Paediatric Rheumatology International Trials Organization (PRINTO) cohort of JDM patients off treatment to define criteria for inactive disease. Focusing on available clinical assessment tools, they found that three of the following four criteria best identified inactive disease: creatine kinase 150 U/l or less, childhood myositis assessment score (CMAS) at least 48, physician global assessment (PGA) 0.2 or less and manual muscle testing (MMT)-8 score of 78 or less [17^▪^]. It is of note that three of these four criteria focus on muscle disease activity, which may underestimate inflammation in other organs, such as skin and gut.

Several groups have investigated whether the addition of MRI can improve the assessment of patients with JDM. Ladd *et al.*[18] examined the prognostic value of pre-treatment MRI scans of the pelvic and thigh muscle. The overall severity of MRI changes did not correlate with outcome, nor did the extent of muscle involvement, but signal abnormality in the subcutaneous fat was a specific (but not sensitive) indicator of an aggressive disease course [18]. In a separate study [19], whole body MRI (WB-MRI) was used to assess inflammation of muscle, fascia and subcutaneous fat in 41 JDM patients at presentation and repeated in 18 patients at a median follow-up of 9 months. As expected, WB-MRI scores strongly correlated with MMT-8 and CMAS, but more interestingly, at follow-up, MRI identified more patients in remission (defined by PRINTO criteria [17^▪^]) than MMT. This may reflect alternative explanations for muscle weakness such as steroid atrophy or disuse, than are mistakenly attributed to disease activity. Further larger-scale studies are needed to confirm the utility and cost-effectiveness of MRI in clinical practice and agree a common standard for scoring of MRIs, which currently varies between centres [20].

### Therapy

The year 2013 witnessed the publication of the first double-blind randomized controlled trial (RCT) of a therapeutic agent in IIMs, and reflects a major achievement of collaboration across the disciplines of paediatrics and adult medicine, rheumatology and neurology. This trial examining the efficacy of the anti-CD20 antibody, rituximab, was conducted in both adult (76 each of polymyositis and dermatomyositis) and childhood IIMs (48 JDM), and carried out across 31 sites in the US and Canada [21^▪▪^]. To limit the duration of the placebo phase of the trial, investigators undertook a staggered crossover design, with an 8-week delay between ‘early’ and ‘late’ rituximab-treated groups. On the basis of anecdotal clinical data, results from trials of SLE, and the prompt clearance of circulating B cells after drug treatment with rituximab, it was assumed that the drug would have a rapid onset of action. Unfortunately, the trial did not meet its primary end point to detect a difference in the time to reach definition of improvement (DOI) in the early vs. late arms. This result may have reflected a slower and less potent effect of rituximab than expected. However, there was encouraging news for paediatric rheumatologists, as patients with JDM showed a much greater treatment effect between early and late groups when compared with adult IIMs, but there were insufficient numbers to detect a significant difference. Infections were the commonest serious adverse events, including pneumonia in six, cellulitis in six, urosepsis in two and herpes zoster in two. In conclusion, more studies are needed before rituximab can be recommended as a rescue therapy for JDM.

A retrospective case series showed encouraging results for the use of mycophenolate mofetil (MMF) in 12 patients with JDM [22]. In a retrospective controlled study [23] of JDM, intravenous immunoglobulin (IVIG) was found to reduce disease activity, particularly in steroid-resistant patients.

Physical therapy is a routine part of the rehabilitation of IIMs in most specialist units, yet lacks a strong evidence base. A new RCT to test the efficacy of a 12-week home-based exercise programme and see if this improves aerobic exercise capacity, isometric muscle strength and perception of fatigue is ongoing [24].

With limited evidence to inform treatment decisions in JDM, the dosing and duration of corticosteroid treatment and use of rescue therapies for steroid-resistant patients vary widely between centres. Over recent years, the North American Childhood Arthritis and Rheumatology Research Alliance (CARRA) have carried out consensus conferences to develop standardized treatment guidelines for JDM. In their latest publication, CARRA defines the clinical characteristics of patients with JDM of moderate severity, and for these patients, suggests a corticosteroid treatment regime beginning at 2 mg/kg, weaning to 1 mg/kg by 14 weeks, and stopping at 50 weeks [25^▪▪^]. Although the rate of steroid weaning may be slow for those patients who respond rapidly to treatment, overall these guidelines offer a useful synthesis of expert opinion across the major international centres that treat JDM.

### Prognosis and long-term outcomes

Long-term functional data in adult patients who had JDM as children are still scarce; therefore, two recent studies in such adults are important and both suggest that functional outcomes for these patients are far from optimal. A study [26^▪^] of 39 adults who had JDM, assessed at a median of 22 years post-disease onset, showed that these patients had reduced quality of life compared with age-matched controls. A second study [27] assessed aerobic fitness, in 36 JDM patients at 2–36 years after onset, and found that 67% had reduced fitness as measured by maximal oxygen uptake (VO_2max_) as a measure of muscle function.

Although validated biomarkers with which to predict outcomes are still lacking, progress has been made in markers of clinical phenotypes, including the clear demonstration that myositis-specific autoantibodies correlate with specific clinical features. Analysis in a large US cohort has confirmed that the juvenile IIMs are heterogeneous and can be clinically divided by autoantibody status [28^▪^]. The presence of anti-nuclear matrix protein (NXP)-2 antibodies has recently been shown to be associated with calcinosis in children with JDM [29^▪^].

Two recent studies have investigated how age at onset may affect disease type, features and prognosis of JDM. In the first, children whose disease manifested before the fifth birthday were seen to have more ulcerative skin disease and oedema, and a lower incidence of sclerodermatous overlap features [30]: children with a younger age at onset were treated more aggressively but achieved comparable rates of remission by 2 years after diagnosis. The second study [31] found that children whose JDM starts before the age of 3 years had a milder disease course but more atypical features.

## CONCLUSION

In conclusion, in recent years, work in the juvenile myopathies specifically, as well as studies that have combined both adults and children, have led to many novel insights and developments in our understanding. Clinical trials in this rare disease are challenging, but the increasingly well organized collaborative networks should facilitate these much-needed studies in the future.

## Acknowledgements

*The authors would like to thank the families and patients who have generously agreed to be part of the UK JDM Cohort and Biomarker study (**http://www.juveniledermatomyositis.org.uk**), and all the contributors to the UK Juvenile dermatomyositis Research Group.*

### Conflicts of interest

K.N. is a Wellcome Trust Intermediate Clinical Fellow, ref 097259 L.W. is supported by grants from the Myositis Support Group, Wellcome Trust, (085860) Great Ormond Street Children's Charity and Arthritis Research UK (20164).

There are no conflicts of interest.

## REFERENCES AND RECOMMENDED READING

Papers of particular interest, published within the annual period of review, have been highlighted as:

▪ of special interest
▪▪ of outstanding interest
